# Lippincott’s New Medical Dictionary

**Published:** 1911-03

**Authors:** 


**Lippincott's New Medical Dictionary.** By **Henry W. Cattell, A.M.** M.D., Editor of International Clinics. Octavo, pp. 1108. Illustrated. Philadelphia and London: J. B. Lippincott Co. (Limp leather, thumb index, $5.00.)

This is one of the latest, if not the very latest, of the works in its line. It is a marvel of completeness and apparently contains the last new word in medical lore.

By a carefully elaborated and original system of condensation and the omission of unessentials it is believed the present work contains a larger number of actual entries than any other of corresponding size. To aid further in this space-saving arrangement, words having a common etymological element, especially of a medical nature, are usually grouped together. In the definitions of words the aim has been to keep them as concise as is consistent with clearness and accuracy. Considerable matter of an encyclopedic and descriptive nature appears; under the chief diseases some account of their symptoms, etiology and treatment; under drugs their action, therapeutic use and dose, the latter in both the metric system and common equivalent; and under anatomical parts an outline of their structure and function. There are frequent references to books and journals, and under the more important proper names the dates of birth and death and the nationality are given. An extremely ingenius and simple system of cross-references has been adopted which is one of the distinguishing and most valuable features of the volume.

